# Selective Immunomodulation of Inflammatory Pathways in Keratinocytes by the Janus Kinase (JAK) Inhibitor Tofacitinib: Implications for the Employment of JAK-Targeting Drugs in Psoriasis

**DOI:** 10.1155/2018/7897263

**Published:** 2018-11-19

**Authors:** Martina Morelli, Claudia Scarponi, Laura Mercurio, Francesco Facchiano, Sabatino Pallotta, Stefania Madonna, Giampiero Girolomoni, Cristina Albanesi

**Affiliations:** ^1^Section of Dermatology, Department of Medicine, University of Verona, Verona 37126, Italy; ^2^Laboratory of Experimental Immunology and V Division of Dermatology, Istituto Dermopatico dell'Immacolata, IDI-IRCCS, Rome 00167, Italy; ^3^Department of Oncology and Molecular Medicine, Istituto Superiore di Sanità (ISS), Rome 00161, Italy

## Abstract

IFN-*γ* and IL-22 are deeply involved in the pathogenesis of psoriasis, as they boost the expression of inflammatory genes and alter proliferative and differentiative programs in keratinocytes. The JAK1/JAK2/STAT1 and JAK1/TYK2/STAT3 pathways triggered by IFN-*γ* and IL-22, respectively, are aberrantly activated in psoriasis, as highlighted by the peculiar STAT1 and STAT3 signatures in psoriatic skin lesions. To limit the detrimental consequences of IFN-*γ* and IL-22 excessive stimulation, psoriatic keratinocytes activate suppressor of cytokine signaling (SOCS)1 and SOCS3, which in turn dampen molecular signaling by inhibiting JAK1 and JAK2. Thus, JAK targeting appears to be a reasonable strategy to treat psoriasis. Tofacitinib is an inhibitor of JAK proteins, which, similarly to SOCS, impedes JAK phosphorylation. In this study, we evaluated the immunomodulatory effects of tofacitinib on epidermal keratinocytes in *in vitro* and *in vivo* models of psoriasis. We demonstrated the selectivity of tofacitinib inhibitory action on IFN-*γ* and IL-22, but not on TNF-*γ* or IL-17 proinflammatory signaling, with suppressed expression of IFN-*γ*-dependent inflammatory genes, and restoration of normal proliferative and differentiative programs altered by IL-22 in psoriatic keratinocyte cultures. Tofacitinib also potently reduced the psoriasiform phenotype in the imiquimod-induced murine model of psoriasis. Finally, we found that tofacitinib mimics SOCS1 or SOCS3 activities, as it impaired the same molecular pathways in IFN-*γ* or IL-22-activated keratinocytes.

## 1. Introduction

Psoriasis is an immune-mediated skin disease characterized by epidermal abnormalities and prominent inflammatory cell infiltrate [1–3]. Current opinion on the pathogenesis of psoriasis emphasizes the role of cytokine signaling to drive an inflammatory cycle, in which infiltrating dendritic cells and autoreactive T lymphocytes, mainly represented by IL-17-producing T cells, T-helper-1 (Th1), and Th22 cells, release IL-17, IFN-*γ*, IL-22, and TNF-*α*. All these cytokines induce keratinocyte expression of a plethora of immune mediator determinant for recruitment and activation of additional dendritic cells and T lymphocytes, which in turn reinforce the pathogenic cycle by perpetuating keratinocyte activation [4–6]. Proinflammatory cytokines, in particular, IL-22 and IL-17, are also responsible for hyperproliferation and altered terminal differentiation of keratinocytes, as well as impairment of the apoptotic pathways, all typical features of psoriasis [4–6]. Thus, IFN-*γ* and IL-22 inflammatory cytokines are deeply involved in the pathogenesis of psoriasis, and their Janus Kinase (JAK)1/JAK2/signal transducers and activators of transcription (STAT)1 and JAK1/tyrosin kinase (TYK)2/STAT3 proximal signaling are aberrantly activated, as highlighted by STAT1 and STAT3 signatures in psoriatic skin lesions [7–10]. Also, IL-17 and TNF-*α* proinflammatory cytokines elicit immune responses in psoriatic keratinocytes, through molecular pathways independent on JAK/STAT and involving NF-*κ*B, Act1, and ERK1/2 [11, 12].

To limit an excessive stimulation by inflammatory cytokines, keratinocytes express suppressors of cytokine signaling (SOCS) molecules, a family of endogenous inhibitors of cytokine-dependent signaling [6, 13–16]. SOCS1 and SOCS3 function as potent suppressors of IFN-*γ* and IL-22 signaling in keratinocytes, respectively. At molecular level, SOCS1 and SOCS3 inhibit JAK1–2 by functioning as pseudosubstrates, hence impeding the activation of IFN-*γ* and IL-22 receptors and downstream STATs. As a consequence of the loss of STAT1 activity, keratinocytes overexpressing SOCS1 can no longer express inflammatory molecules in response to IFN-*γ* [13–15]. Similarly, IL-22-induced proliferative and antidifferentiative effects on keratinocytes are efficiently counteracted by SOCS3-dependent STAT3 inhibition [15, 17]. On the other hand, SOCS cannot influence JAK-independent molecular pathways in keratinocytes, including TNF-*γ* signaling [13].

Because of the importance of inflammatory cytokines in psoriasis, JAK targeting represents a logical strategy to treat this disease. Various JAK inhibitors are in preclinical development or have been tested in clinical trials. Among them, tofacitinib is an oral JAK inhibitor with an intracellular mechanism of action against JAKs, already in use for systemic treatment of rheumatoid arthritis [18], and under evaluation for the treatment of both plaque psoriasis [19] and psoriatic arthritis [20]. Phase 3 studies in patients with moderate-to-severe chronic plaque psoriasis have demonstrated the efficacy of tofacitinib in improving clinical outcomes [21]. JAK inhibition by tofacitinib strongly reduces clinical signs of psoriasis, and, potently blocks signaling through the common *γ* chain-containing receptors, including IL-2, IL-4, IL-7, IL-9, and IL-15, or through canonical receptors for cytokines, such as IFN-*γ*, IL-21, IL-6, and to a lesser extent, IL-12 and IL-23 [22]. In preclinical models, tofacitinib was shown to affect both innate and adaptive immune responses and inhibited pathogenic T helper (Th)17 cell differentiation by suppressing IL-23 expression [23].

While the mechanisms of T-cell activity inhibition and modulation of differentiation by tofacitinib are well characterized [23–25], few information on the immunomodulatory effects on psoriasis-related pathways activated in resident keratinocytes, or on its capability to mimic SOCS inhibitory circuits exist for this drug. In this study, we evaluated the immunomodulatory effects of tofacitinib on epidermal keratinocytes in experimental *in vitro* and *in vivo* models of psoriasis. In particular, we studied the tofacitinib effect on JAK/STAT pathway and downstream inflammatory molecules in human keratinocyte cultures activated with proinflammatory molecules related to psoriasis, including IFN-*γ*, IL-22, IL-17, and TNF-*γ*, as well as *in vivo* in the imiquimod- (IMQ-) induced murine model of psoriasis. We also investigated the impact of tofacitinib on other protein targets induced by IFN-*γ* or IL-22 signaling in keratinocytes and to mimic SOCS1 or SOCS3 activities.

## 2. Materials and Methods

### 2.1. Keratinocyte Cultures and Treatments

Primary cultures of human keratinocytes were obtained from skin biopsies of psoriatic patients (*n* = 5) afferent to 5th Dermatology Unit at IDI-IRCCS and prepared as previously described [6]. Patients had definite plaque-type psoriasis diagnosed according to standard criteria, and they had not received any systemic or topical therapy for at least 1 month before skin donation. Skin biopsies were obtained after patient's informed written consent, with the approval of the IDI-IRCCS Local Ethics Committee (Prot. N. 474/1/2016; study: “Studio delle chinurenine in pazienti affetti da psoriasi”). Second- or third-passage keratinocytes were used in all experiments, with cells cultured in the serum-free medium KGM (Clonetics, San Diego, CA), for at least 3–5 days (at 60–80% confluence) before performing treatments. Some experiments were performed on keratinocyte cultures undergoing terminal differentiation, achieved by growing cells at 100% of confluence (*t*0) and, thus, keeping them in culture for another 4 d.

Stimulations with 200 U/ml recombinant human (rh) IFN-*γ* (R&D Systems, Minneapolis, MN, USA), as well as 50 ng/ml rh TNF-*α*, IL-22, or IL-17 (all from R&D Systems), were performed in keratinocyte basal medium (KBM, Clonetics). Tofacitinib (CP 690,550 compound) was obtained from Pfizer Inc. (Peapack, NJ) and administered by pretreating cultures for 1 h before cytokine stimulation. Cytotoxicity of tofacitinib was previously tested by measuring the activity of lactate dehydrogenase (LDH) released from keratinocyte cultures, using Cytotoxicity Detection Kit Plus-LDH (Roche Diagnostics, Milan, Italy), following the manufacturer' instructions.

### 2.2. Immunoprecipitation, Immunoblotting, and Densitometry

Protein extract preparation, immunoprecipitation, and immunoblotting were performed accordingly to standard procedures [6]. The Abs used for the study were as follows: anti-IFN-*γ*R*α* subunit (C-20), anti-IL-22R1 (3-RE40), anti-TYK2 (C-20) (all from Santa Cruz Biotechnology, Santa Cruz, CA, USA), anti-phosphotyrosine (clone 4G10; Upstate Biotechnologies, Temecula, CA), anti-JAK1, anti-JAK2 (Upstate Biotechnologies), anti-phosphotyrosine- (pTyr701-) STAT1 (Santa Cruz Biotechnology), anti-phosphoserine- (pSer727-) STAT1, anti-phosphotyrosine- (pTyr705-) STAT3 and anti-phosphoserine- (pSer727-) STAT3 (Cell Signaling), anti-STAT1 and anti-STAT3 (C-20) (Santa Cruz Biotechnology), anti-phospho-ERK1/2 (E4; Santa Cruz Biotechnology), anti-ERK1/2 (C-16; Santa Cruz Biotechnology), anti-phospho-p65 (Ser276), anti-I*κ*B*α*, HRP-conjugated anti-c-myc (9E10), anti-p63 (4A4), anti-*β*-actin (all from Santa Cruz Biotechnology, Santa Cruz, CA, USA), anti-keratin (KRT)1, and anti-loricrin (both from Covance, Meryville, CA). Filters were properly developed with anti-mouse, anti-goat, or anti-rabbit Ig Abs conjugated to HRP using the ECL-plus detection system (Amersham, Dubendorf, Switzerland) or, otherwise, the SuperSignal West Femto kit (Pierce, Rockford, IL, USA). Immunoblots of experiments were subjected to densitometry using an Imaging Densitometer model GS-670 (Bio-Rad) supported by the Molecular Analyst Image software, and band intensities were evaluated in three independent experiments. Data are expressed as fold induction ± SD in experimental time-course relative to untreated or tofacitinib-treated samples, to which were given a value of 1.

### 2.3. Transient Transfection and Luciferase Assay

Cultured keratinocytes grown in six-well plates were transiently transfected with the STAT3-responsive plasmid pLucTKS3 (a generous gift of Prof. J. Turkson, University of Central Florida, Orlando, FL) or pGASLuc plasmid by using Lipofectin reagent (Invitrogen), according to the manufacturer's instructions. At 24 h post transfection, cells were pretreated with tofacitinib for 1 h and then stimulated with IL-22 or IFN-*γ* for 8 h. After cell lysis in an appropriate buffer (Promega Italia, Milan, Italy), *Firefly* luciferase activity was measured using Dual-Glo Luciferase Assay System (Promega). To normalize the transfection efficiency, pRL-null plasmid encoding the *Renilla* luciferase was included in each transfection. Luciferase activity was further normalized by total cellular protein content assayed using Bradford (Sigma-Aldrich, Milan, Italy).

### 2.4. Intracellular Signaling Array

PathScan Intracellular Signaling Array Kit was purchased from Cell Signaling Technology (Cell Signaling Technology, Beverly, MA; Catalog #7323). This array allows the simultaneous detection of 18 signaling molecules when phosphorylated or cleaved. They include ERK1/2, STAT1, STAT3, Akt (Thr308 and Ser473 phosphorylation), AMPKa, S6 ribosomal protein, mTOR, HSP27, Bad, p70 S6 kinase, PRAS40, p53, p38, SAPK/JNK, PARP, caspase 3, and GSK-3b. Whole protein lysates from keratinocyte cultures treated with IFN-*γ*, IL-22, or TNF-*α* in the presence or absence of tofacitinib were prepared using lysis buffer that was provided in the kit and processed following the manufacturers' instructions. The Bio-Rad Gel Documentation System was used to take detailed pictures of the array using the Quantity One software using the ChemiDoc XRS function. Values of graphs are expressed as densitometric units and were normalized to internal positive control.

### 2.5. Proliferation Assays

8 × 10^4^ cells were seeded in 12-well plates and, the day after, starved in KBM. Culture stimulation with IFN-*γ*, IL-22, or TNF-*α* was conducted either in the presence or absence of tofacitinib. After 2 d of treatment, cells were evaluated by Trypan blue exclusion test. Crystal violet assays were also performed to evaluate proliferation. Thus, 2 × 10^4^ cells were grown for 48 h in 96-well plates and stained with 0.5% crystal violet, whose incorporation was measured at 540 nm in an ELISA reader (model 3550 UV ELISA reader; Bio-Rad, Hercules, CA).

### 2.6. Apoptosis Analysis

Apoptosis of keratinocytes was evaluated using the FITC Annexin V/propidium iodide (PI) apoptosis detection kit (BD Biosciences, Milan, Italy). Viability, necrosis, and apoptosis were analysed by flow cytometry. Cells were analysed with a FACScan equipped with Cell Quest software. The percentage of Annexin V^+^, PI^+^, and Annexin V/PI^+^ cell populations were evaluated in keratinocyte cultures left untreated or treated with IFN-*γ*, IL-22, or TNF-*α* in the presence or absence of tofacitinib.

### 2.7. RNA Isolation and Real-Time Polymerase Chain Reaction (PCR)

Total RNA from keratinocyte cultures was extracted using the TRIzol reagent (Invitrogen); mRNA was reverse-transcribed into cDNA and analysed by real-time PCR. The expression of human SOCS3, S100A7, IL-20, HBD-2, LL-37, and HPRT-1 mRNA was evaluated in the ABI Prism SDS 7000 PCR instrument (Applied Biosystems, Branchburg, NJ), using SYBR Green PCR reagents or TaqMan PCR Master Mix. The same PCR tools were employed to analyse murine IL-17A, IL-22, IFN-*γ*, TNF-*α*, CXCL10, CCL2, CCL20, CXCL16, and IL-6 mRNAs. The forward and reverse primers employed for real-time PCR for SOCS3 were 5′-AAGGACGGAGACTTCGATTCG-3′ and 5′-AAACTTGCTGTGGGTGACCAT-3′, and for LL-37 5′-TTTTGCGGAATCTTGTACCCA-3′ and 5′-TCTCAGAGCCCAGAAGCCTG-3′. The sequences of the primers for *β*-defensin- (HBD-) 2 mRNA have been previously described [26]. Primers for S100A7, IL-20, and HPRT-1 were provided by Applied Biosystems (HS 00161488, HS 00218888, and HS 4333768, respectively). Primers used for the detection of murine molecules were retrieved from previous studies [27]. Human and murine mRNA level values were normalized to HPRT-1 and *β*-2-microglobulin mRNA, respectively. The values obtained from triplicate experiments were averaged, and data presented as mean 2^−ΔΔCT^ ± SD.

### 2.8. Multiplex Immunoassay and ELISA

Media conditioned for 48 h by psoriatic keratinocyte cultures stimulated with IFN-*γ* or IL-22 in the presence or absence of tofacitinib were harvested and filtered. The simultaneous quantitative measurement of cytokines/chemokines in small amounts of supernatants was achieved by using the xMAP multiplex technology (Luminex) and a BioPlex 200 System equipped with magnetic washer workstation Bio-Plex ProTM and Manager Software version 6.1 (Bio-Rad Laboratories, Milan, Italy). In particular, a Pro-Human Cytokine Panel (Bio-Plex, Pro-Human Chemokine 40-plex Panel, cat # 171AK99MR2, Bio-Rad) was used to measure the following analytes: 6Ckine/CCL21, BCA-1/CXCL13, CTACK/CCL27, ENA-78/CXCL5, Eotaxin/CCL11, Eotaxin-2/CCL24, Eotaxin-3/CCL26, Fractalkine/CX3CL1, GCP-2/CXCL6, GM-CSF, Gro-*α*/CXCL1, Gro-*β*/CXCL2, I-309/CCL1, IFN-*γ*, IL-1*β*, IL-2, IL-4, IL-6, IL-8/CXCL8, IL-10, IL-16, IP-10/CXCL10, I-TAC/CXCL11, MCP-1/CCL2, MCP-2/CCL8, MCP-3/CCL7, MCP-4/CCL13, MDC/CCL22, MIF, MIG/CXCL9, MIP-1*α*/CCL3, MIP-1*δ*/CCL15, MIP-3*α*/CCL20, MIP-3*β*/CCL19, MPIF-1/CCL23, SCYB16/CXCL16, SDF-1*α* + *β*/CXCL12, TARC/CCL17, TECK/CCL25, and TNF-*α*, following the manufacturers' instructions. In parallel, CCL5 was measured with a commercially available sandwich ELISA kit (R&D Systems) and an ELISA reader model 3550 UV (Bio-Rad). Psoriatic keratinocyte cultures were conducted in duplicate using two different keratinocyte strains. Data were expressed as mean pg/ml or ng/ml ± SD.

### 2.9. IMQ-Induced Psoriasiform-Like Model

8 weeks old female BALB/cJ mice (Harlan Laboratories, San Pietro al Natisone, Italy) were employed in all the experiments. Shaved mouse dorsal skin was treated daily for 5 consecutive days with 50 mg Aldara cream containing 5% IMQ (MEDA AB, Solna, Sweden). On day 5, full-thickness skin biopsies of the treated area were collected with an 8 mm biopsy puncher. Skin was fixed in neutral buffered formalin (Sigma-Aldrich, St. Louis, MO, USA) for histopathological analysis. In some experiments, 50 *μ*l Aldara cream was mixed with tofacitinib (in DMSO solution) at a final concentration of 10 and 0.5 mM. A group of 10 mice was used for each experimental condition. On day 5, full skin was fixed in neutral buffered formalin (Sigma-Aldrich, St. Louis, MO, USA) for histopathological analysis. Otherwise, full skin was frozen in nitrogen liquid and further processed for RNA extraction, which was performed by using TRIzol reagent (Invitrogen). RNA from ten mice per experimental group were pooled, reverse-transcribed into cDNA, and analysed by real-time PCR, as previously described.

### 2.10. Histopathology and Immunohistochemistry

Fixed murine skin was embedded in paraffin, and tissue sections were deparaffinized and stained with H&E for histological analysis. Epidermal and scale thickness and cell infiltrate number were analysed as parameters of skin acanthosis and inflammation. Average epidermal and scale thickness was quantified by a researcher blind to the experimental groups who took five measurements per three sections for each mouse. Cells infiltrating dermis were also counted in three skin sections for each mouse. Immunohistochemistry was performed by using primary Abs against CD3 (Dako, Glostrup, Denmark), Ly6G, CD11c, and CD11b (BD Biosciences), Ki67 (Novocastra, Newcastle upon Tyne, UK), KRT10 (Covance), phospho-STAT3 (Tyr705) and phospho-STAT1 (Tyr701) (both from Cell Signaling), IL-17A (R&D Systems) and IL-22 (Novus Biologicals, Oakdille, Canada), and immunoreactivities developed with secondary biotinylated mAbs and staining kits (Vector Laboratories, Burlingame, CA, USA). Sections were counterstained with Mayer's hematoxylin and were visually analysed by two pathologists experienced in dermatology. Positivity was evaluated in 5 adjacent fields at a magnification of 200x. A semiquantitative, four-stage scoring system was applied, ranging from negative immunoreactivity (0) to strong immunoreactivity (4+) for KRT10 in the epidermis.

### 2.11. Stable Keratinocyte Transfectants

HaCaT cells were stably transfected with *myc*/SOCS1, *myc*/SOCS2, *myc*/SOCS3, or empty pcDNA3 (mock) plasmids as previously reported [6, 13]. HaCaT SOCS clones were treated with IFN-*γ* or IL-22, whereas mock clones were pretreated with tofacitinib and then stimulated with IFN-*γ* or IL-22 in DMEM.

### 2.12. Statistical Analysis

For *in vitro* studies, statistical significance was evaluated using Wilcoxon's signed rank test (SigmaStat; Jandel, San Rafael, CA, USA). Values of *p* ≤ 0.05 were considered significant. For *in vivo* experiments, the significance of differences between experimental groups (mice treated with IMQ vs. mice treated with IMQ plus tofacitinb 100 mM or 5 mM) was calculated by unpaired Student's *t*-test. Statistical analysis was performed with Prism v.5.0 (GraphPad Software, La Jolla, CA, USA), and values are expressed as the mean + SD of *n* animals. Values of *p* < 0.05 were considered significant.

## 3. Results

### 3.1. Tofacitinib Efficiently Inhibits on JAK/STAT-Dependent Pathways in IFN-*γ*- or IL-22-Activated Keratinocytes

**Figure 1:**
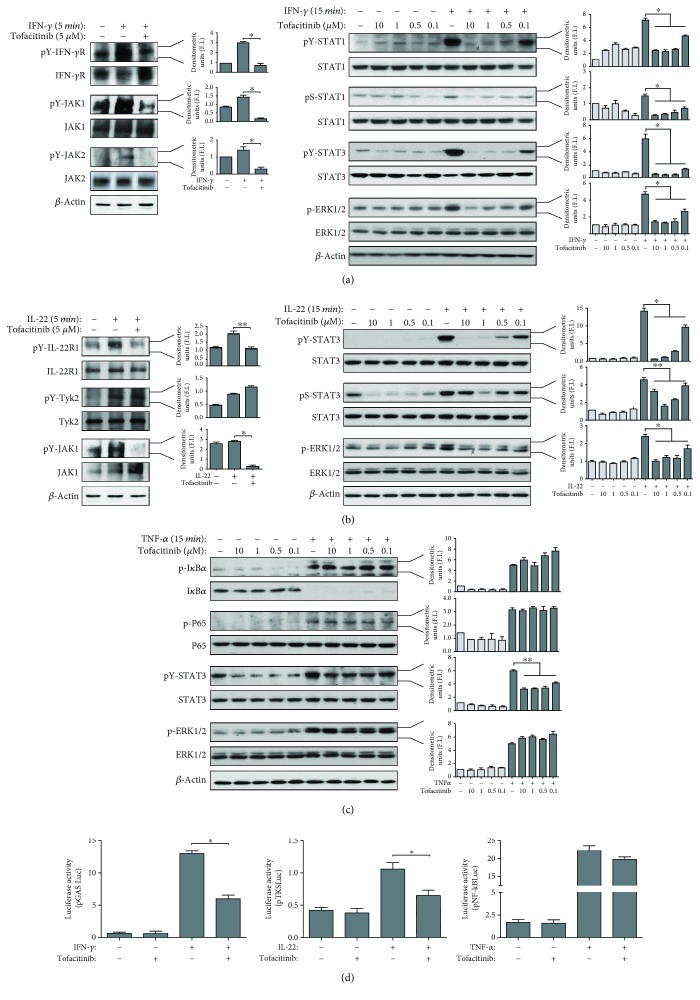
Tofacitinib inhibits IFN-*γ*- and IL-22- but not TNF-*α*-induced molecular signaling in psoriatic keratinocytes cultures. (a) Protein extracts obtained from psoriatic keratinocytes pretreated or not with vehicle alone or the indicated doses of tofacitinib, and then stimulated or not with IFN-*γ* for the indicated time periods, were subjected to immunoprecipitation for IFN-*γ*R*α*, JAK1, or JAK2 and Western blotting analysis by using anti-phosphotyrosine Ab to detect IFN-*γ*R*α*, JAK1, or JAK2 phosphorylation. Filters were stripped and reprobed with anti-IFN-*γ*R*α*, JAK1, or JAK2 Abs. Phosphorylated and unphosphorylated forms of STAT1, STAT3, and ERK1/2 were monitored in keratinocytes by WB analysis. (b) Protein extracts were obtained from keratinocytes pretreated or not with tofacitinib in the presence of IL-22 for the indicated time periods and were subjected to immunoprecipitation for IL-22R1, TYK1, or JAK2 and, then, WB analysis by using anti-phosphotyrosine Ab to detect IL-22R1, TYK1, or JAK2 phosphorylation. Filters were stripped and reprobed with anti-IL-22R1, TYK1, or JAK2 Abs. Phosphorylated and unphosphorylated forms of STAT1, STAT3, and ERK1/2 were also monitored in keratinocytes by WB analysis. (c) Protein extracts were obtained from keratinocytes pretreated or not with tofacitinib in the presence of TNF-*α* for 15 min and then analysed by WB analysis to detect basal or phospho-I*κ*B*α*, basal or phosphor-p65 subunit of NF-*κ*B, and phosphorylated and unphosphorylated forms of STAT3 and ERK1/2. Graphs in (a), (b), and (c) represent densitometric analyses of the indicated proteins shown in representative WB. Data are expressed as mean ± SD fold induction (F.I.) calculated relatively to the untreated samples, which were given a value of 1. ^∗^*p* < 0.01, ^∗∗^*p* < 0.05. (d) Psoriatic keratinocyte cultures transiently transfected with a STAT1-, STAT3-, or NF-*κ*B-responsive plasmids, termed, respectively, pGAS-Luc, pLucTKS3, or pNF-*κ*B-Luc were treated with 5 *μ*M tofacitinib or vehicle alone for 2 h and then stimulated with IFN-*γ*, IL-22, or TNF-*α* for 8 h prior to assay Firefly luciferase activity on cellular extracts. Data are expressed as Firefly luciferase values normalized to Renilla luciferase and micrograms of proteins and are shown as mean + SD of *n* = 6 samples pooled from two experiments. ^∗^*p* < 0.01.

We initially studied the impact of tofacitinib on intracellular pathways activated in human keratinocytes by proinflammatory cytokines with a pathogenic role in psoriasis, including IFN-*γ*, IL-22, TNF-*α*, and IL-17. To this end, primary keratinocyte cultures were established from skin biopsies of psoriatic patients (*n* = 5). The choice to employ psoriatic keratinocyte cultures raised from the fact that these strains are more responsive to triggering factors, as compared to keratinocytes obtained from healthy donors, probably due to their genetic background and different susceptibilities to proinflammatory cytokines [4, 5]. Tofacitinib had no cytotoxic effects on keratinocytes even at higher concentrations, as tested by measuring the activity of lactate dehydrogenase released by cultures (not shown). One hour pretreatment with different doses of tofacitinib (0.1–10 *μ*M) was followed by stimulation of keratinocyte cultures with rh IFN-*γ* (200 U/ml), IL-22 (50 ng/ml), TNF-*α* (50 ng/ml), or IL-17 (50 ng/ml) for different time periods (Figure 1, data not shown). Tofacitinib efficiently inhibited IFN-*γ* and IL-22 proximal signaling, with reduced phosphorylation of IFN-*γ* R, JAK1, and JAK2, as well as IL-22R1 and JAK1, but not TYK2, respectively (Figures 1(a) and 1(b), left). As a consequence of proximal signaling inhibition, downstream STAT1 and STAT3 phosphorylation was dose dependently inhibited in IFN-*γ*-stimulated cultures (Figure 1(a), right). Interestingly, phospho-ERK1/2 activation was also reduced by tofacitinib, even if less potently if compared to STATs (Figure 1(a), right). Similarly to what we observed for IFN-*γ*, IL-22 could not induce STAT3 or ERK1/2 phosphorylation in the presence of tofacitinib (Figure 1(b), right). In order to evaluate the specificity of action of tofacitinib on pathways dependent on JAKs, we analysed its effects on the signaling of TNF-*α* or IL-17, cytokines which notoriously can activate NF-*κ*B and MAP kinases but not JAKs. As shown in Figure 1(c), tofacitinib could not influence phosphorylation of I*κ*B*α* or p65 NF-*κ*B subunit, nor ERK1/2 in response to TNF-*α*. In contrast, tofacitinib reduced STAT3 activation induced by TNF-*α*, even if at a lower degree if compared to that observed for IFN-*γ*- or IL-22-treated samples. Of note, tofacitinib could not regulate signaling pathways activated by IL-17 in keratinocytes, including NF-*κ*B (data not shown). Finally, tofacitinib potently reduced the IFN-*γ*- or IL-22-induced transactivation of STAT1- or STAT3-binding promoters in keratinocytes, as assessed in cultures transfected with the IFN-*γ*-inducible reporter plasmid, pGAS-Luc, or the IL-22-inducible reporter plasmid, pLucTKS3, respectively (Figure 1(d)). As a whole, these data demonstrate that tofacitinib totally abrogated JAK/STAT pathways activated by IFN-*γ* and IL-22 in human psoriatic keratinocytes, whereas it could not influence JAK-independent molecular pathways, such as those activated by TNF-*γ* or IL-17.

### 3.2. Analysis of the Effect of Tofacitinib on Additional Molecular Pathways Induced by IFN-*γ* or IL-22 in Keratinocyte Cultures

**Figure 2:**
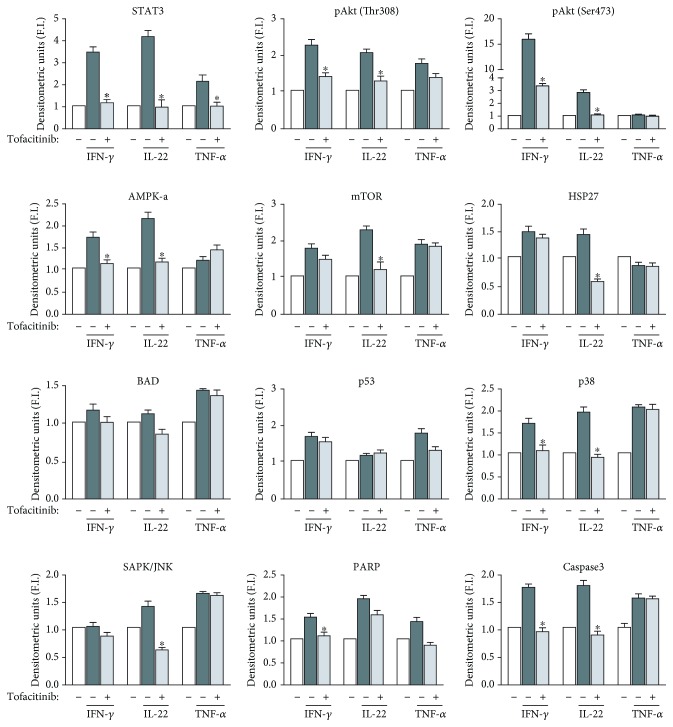
Tofacitinib regulates IFN-*γ*- and IL-22- but not TNF-*α*-induced signaling molecules in psoriatic keratinocytes. Protein extracts obtained from psoriatic keratinocytes pretreated with 5 *μ*M tofacitinib or vehicle alone and then stimulated or not with IFN-*γ*, IL-22, or TNF-*α* for 20 min were used on a PathScan intracellular signaling array, which allows the simultaneous detection of 18 signaling molecules when phosphorylated or cleaved. They include ERK1/2, STAT1, STAT3, Akt (Thr308 and Ser473 phosphorylation), AMPKa, mTOR, HSP27, Bad, p53, p38, SAPK/JNK, PARP, and caspase 3. Developed slides were acquired at ChemiDoc system. Graphs represent densitometric analyses of the indicated proteins. Data are expressed as mean ± SD fold induction (F.I.) calculated relatively to the untreated samples, which were given a value of 1. Protein panel was analysed in two assays with two different keratinocyte strains. Each value was normalized to an internal positive control. ^∗^*p* < 0.05 for samples treated with tofacitinib vs. untreated, in the presence of cytokines.

We next determined the protein phosphorylation profiles of keratinocyte cultures undergoing stimulation with IFN-*γ*, IL-22, or TNF-*α* for 20 min, pretreated or not with tofacitinib. This analysis was performed by using a commercial phospho-kinase array kit (see Materials and Methods), which detects intracellular kinases and signaling node molecules, including Akt, AMPK*α*, mTOR, HSP27, BAD, p53, JNK, p38, PARP, and caspase 3, other than STAT1 and STAT3, specifically activated by IFN-*γ* and IL-22. As shown in Figure 2, tofacitinib could significantly reduce the IFN-*γ*-dependent upregulation of Akt phosphorylation at both Thr308 and Ser473 residues, AMPK*α*, p38, PARP, and caspase 3, other than STAT1 (data not shown) and STAT3. In parallel, it decreased Akt phosphorylation at both Thr308 and Ser473 residues, AMPK*α*, mTOR, HSP27, p38, JNK, and STAT3, activated by IL-22 treatment in keratinocyte cultures (Figure 2). However, tofacitinib inhibition of these additional molecular pathways was weaker if compared to that observed on STAT. Again, TNF-*α*-induced intracellular kinase pattern could not be influenced by tofacitinib treatment, with the exception of STAT3 (Figure 2).

### 3.3. Effects of Tofacitinib on Keratinocyte Proliferation, Differentiation, and Apoptosis Processes

**Figure 3:**
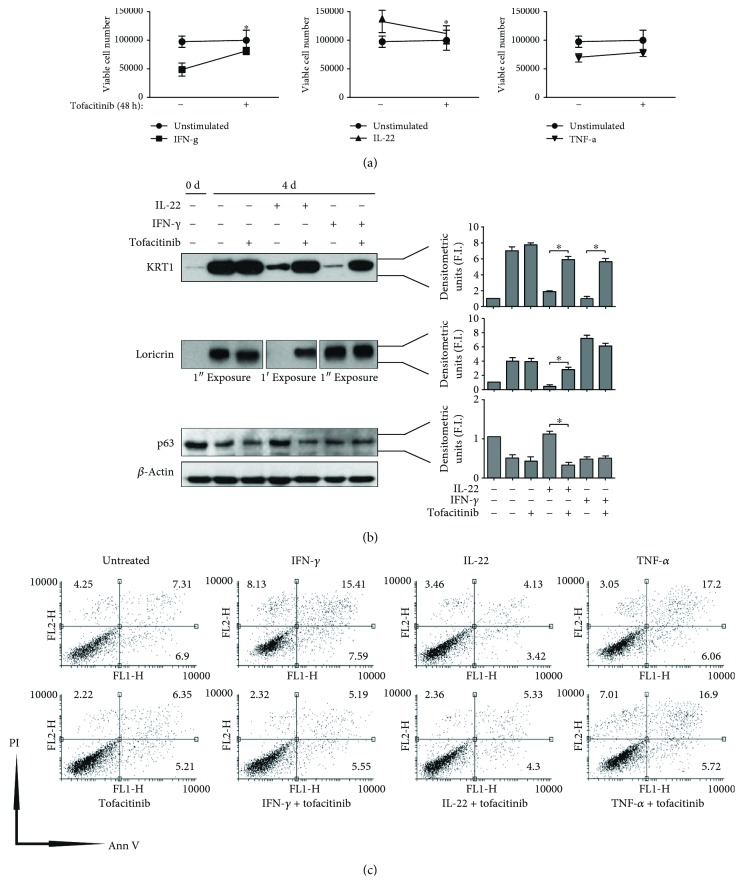
Tofacitinib regulates proliferation, differentiation, and apoptosis of IFN-*γ*- and IL-22- but not TNF-*α*-treated keratinocytes. Proliferation of keratinocyte cultures untreated or treated with 5 *μ*M tofacitinib, either in the presence or absence of IFN-*γ*, IL-22, or TNF-*α*, was analysed by Trypan blue exclusion test (a), which was performed after 48 h of culture. Data are expressed as total cell number ± SD. ^∗^*p* < 0.05. (b) Keratin 1 (KRT1), loricrin, and p63 were analysed by WB by using protein lysates obtained from keratinocyte cultures grown at 100% confluency (0 d) or for additional four days (4 d), in the presence or absence of tofacitinib and IFN-*γ* or IL-22. Graphs show densitometric values ± SD of KRT1, loricrin, or p63. Data are expressed as densitometric units, expressed as fold induction (F.I.) of treated vs. untreated samples, which were given a value of 1. ^∗^*p* < 0.01. (c) Apoptosis of cultured keratinocytes treated with 5 *μ*M tofacitinib in the presence or absence of IFN-*γ*, IL-22, or the proapoptotic stimulus TNF-*α* for 48 h was examined by measuring Annexin (Ann V)/propidium iodide (PI) fluorescence through flow cytofluorimetry. A representative experiment of three performed with two different psoriatic keratinocyte strains is shown, with numbers indicating the percentage of PI^+^ (upper left), Ann V^+^ (lower right), PI/Ann V^+^ (upper right), or negative (lower left) cells.

We assessed whether tofacitinib regulated keratinocyte growth and proliferation, as well as differentiation and apoptosis in psoriatic keratinocyte cultures (*n* = 3). Cells were pretreated with tofacitinib (5 *μ*M) for 1 h and then stimulated with IFN-*γ*, IL-22, or TNF-*α* for 48 h. As previously reported [13, 17], IFN-*γ* decreased proliferation of keratinocyte cultures whereas IL-22 enhanced such process by inhibiting terminal differentiation. When tofacitinib was coadministered, the effects of these cytokines on keratinocyte proliferation were totally reverted (Figure 3(a)). Similarly, tofacitinib could significantly abrogate IL-22-induced inhibition of differentiation, as well as IFN-*γ*-induced apoptosis of keratinocytes (Figures 3(b) and 3(c)). In contrast, tofacitinib did not influence TNF-*γ*-induced processes in keratinocytes, in particular apoptosis, as shown in Figure 3(c).

### 3.4. Regulation by Tofacitinib of Expression of Psoriasis-Related Inflammatory Molecules by Keratinocytes

We then evaluated whether tofacitinib could influence keratinocyte expression of proinflammatory genes induced by IFN-*γ* or IL-22 via JAK/STAT pathway. To this end, the expression of a variety of molecules involved in the induction or control of skin inflammation was studied by cytofluorimetry, bioplex multiplex immunoassays, and real-time PCR analysis of psoriatic keratinocyte cultures pretreated with tofacitinib and then stimulated with rh IL-22 or IFN-*γ*. We found that tofacitinib substantially reduced IFN-*γ*-induced expression of ICAM-1, HLA-DR and MHC class I membrane molecules, and numerous inflammatory mediators, including CX3CL1, CXCL1, CXCL8, CXCL10, CCL1, CCL2, CCL5, MIF chemokines, IL-6, and SOCS3 mRNA (Table 1). Similarly, tofacitinib could downregulate IL-22-induced expression of CX3CL1, CXCL8, CXCL12, and CCL2 chemokines, as well as of IL-20 and SOCS3 in keratinocyte cultures (Table 2). Inflammatory molecules induced by TNF-*α* or IL-17 could not be regulated by tofacitinib (data not shown). Thus, tofacitinib treatment could influence the expression of genes that were regulated in a STAT-dependent manner at the transcriptional level.

### 3.5. Analysis of the Effects of Tofacitinib on the IMQ-Induced Murine Model of Psoriasis

**Figure 4:**
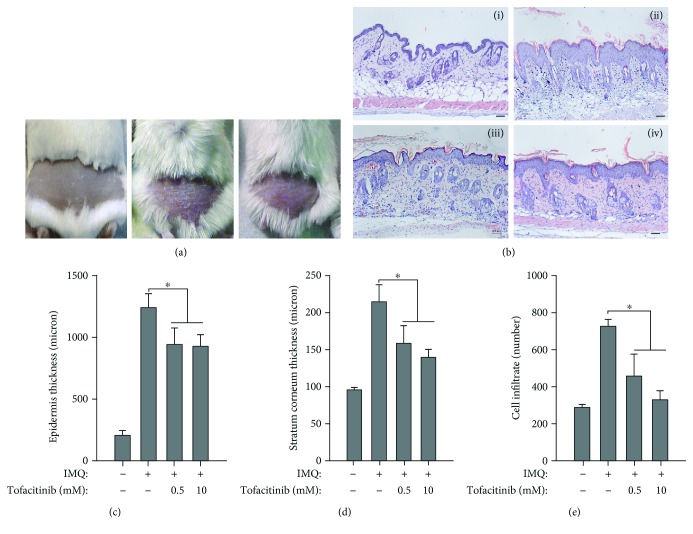
Tofacitinib inhibits inflammatory responses in the IMQ-induced psoriasiform mouse model. (a) Representative pictures of back-shaved mice left untreated (left), IMQ-treated (middle), or undergoing to cotreatment with IMQ and 10 mM tofacitinib. (b) Representative H&E staining of (i) untreated, (ii) treated with IMQ cream, and in the presence of 10 mM (iii) or 0.5 mM (iv) tofacitinib. Mouse skin treated with IMQ reverted their condition after tofacitinib topical application of 0.5 and 10 mM. The quantification of (c) epidermal, (d) scale thickness, and (e) cell infiltrate number was analysed as parameters of skin acanthosis and inflammation. Graphs show means of microns of epidermis and stratum corneum thickness and mean of number of cells infiltrating dermis per section, ±SD *per* group (*n* = 10 mice). ^∗^*p* < 0.001.

**Figure 5:**
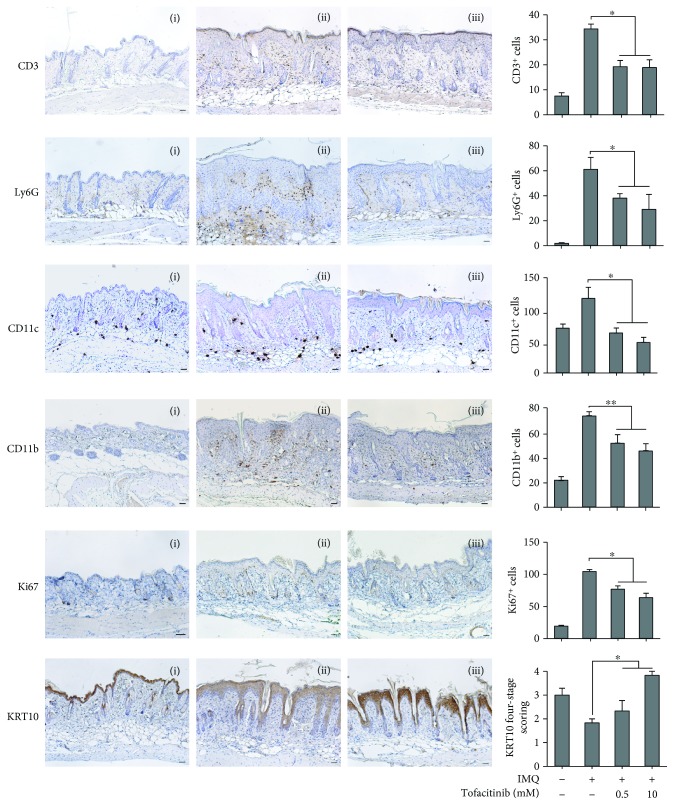
Tofacitinib counteracts IMQ-induced leukocyte infiltration, proliferation, and dedifferentiation in mouse skin. Immunohistochemistry analysis of mouse skin left untreated (i), IMQ-treated (ii), and IMQ-treated in the presence of tofacitinib (iii) shows reduction of positive CD3, LY6G, Ki67, CD11c, and CD11b cells and an increase of KRT10 in the epidermis after tofacitinib treatment. Sections were counterstained with Mayer's hematoxylin and were visually evaluated by a pathologist experienced in dermatology. Bars, 200 *μ*M. One of four representative stainings is shown. Graphs show the mean of number of positive cells or of semiquantitative, four-stage scoring values for KRT10 ± SD *per* three sections *per* experimental group (*n* = 10 mice). ^∗^*p* < 0.01, ^∗∗^*p* < 0.001.

**Figure 6:**
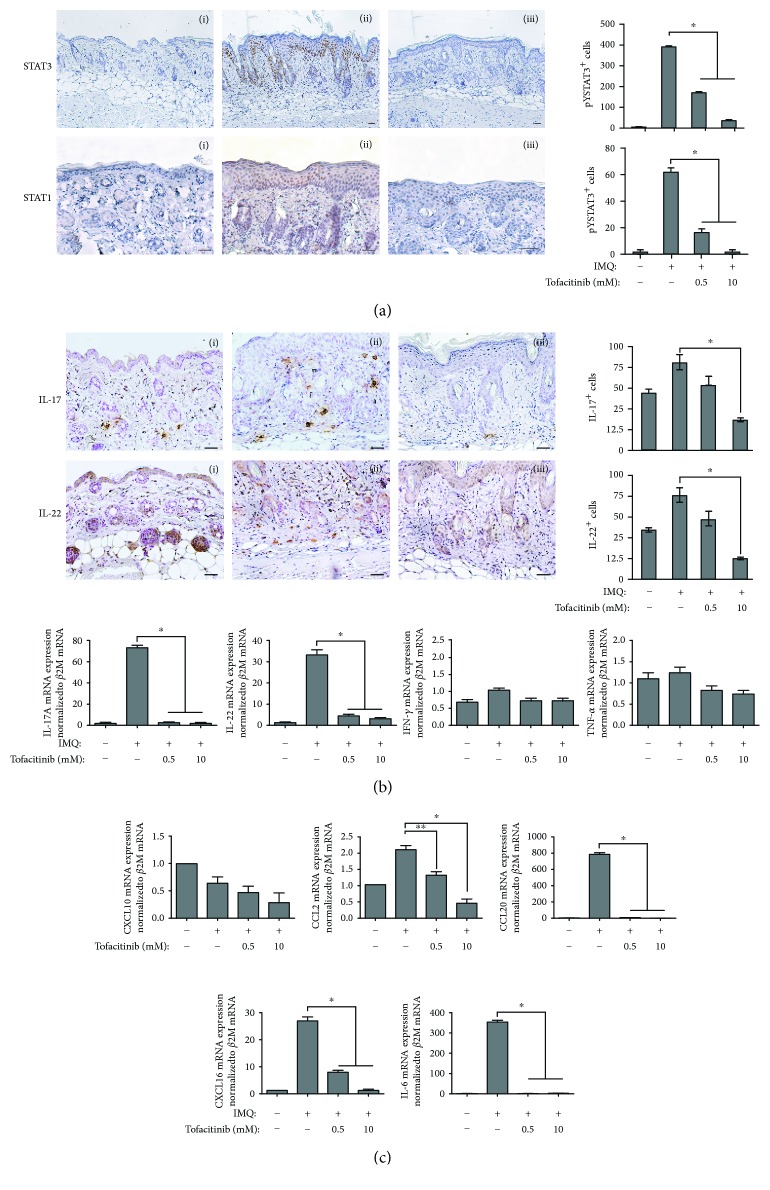
Tofacitinib counteracts IMQ effects in mouse skin. Immunohistochemistry analysis of mouse skin left untreated (i), IMQ-treated (ii), and IMQ-treated in the presence of tofacitinib (iii) shows reduction of STAT1-, STAT3-, IL-17A-, and IL-22-positive cells (a) and (b) after tofacitinib treatment. Sections were counterstained with Mayer's hematoxylin and were visually evaluated by a pathologist experienced in dermatology. Bars, 200 *μ*M. One of four representative stainings is shown. Graphs show the mean number of positive cells ± SD *per* three sections *per* experimental group (*n* = 10 mice). ^∗^*p* < 0.01. In (b), graphs show real-time PCR analyses of IL-17A, IL-22, IFN-*γ*, and TNF-*α* performed on pooled mRNA samples (*n* = 10) of mouse skin treated as indicated. ^∗^*p* < 0.01. In (c), graphs show real-time PCR analyses of CXCL10, CCL2, CCL20, CXCL16, and IL-6 performed on pooled mRNA samples (*n* = 10) of mouse skin treated as indicated. ^∗^*p* < 0.01, ^∗^*p* < 0.05.

IMQ-induced dermatitis in mice can serve as a model for the analysis of pathogenic mechanisms involved in psoriasis [28]. In this model, a major role of the IL-23/IL-17/IL-22 axis has been demonstrated, with IL-22-deficient mice being resistant to psoriasis development induced by IMQ [29]. Thus, the effect of tofacitinib was studied in this model, with the drug being administered together with IMQ for 5 days, at two different concentrations (0.5 and 10 mM). As shown in Figure 4, tofacitinib substantially reverted the psoriasiform phenotype in IMQ-treated mice (Figure 4(a)) and, even at the lower dose, reduced psoriasiform signs, including epidermal and scale thickness, as assessed by quantifying the average of these parameters on images of skin sections stained by H&E (Figures 4(b)–4(d)). Tofacitinib also reduced the widespread inflammatory infiltrate in the dermis, as compared with control (Figures 4(b) and 4(e)). Moreover, tofacitinib administration led to a dose-dependent reduction of the number of CD3^+^ T lymphocytes, Ly6G^+^ neutrophils, CD11c^+^ dendritic cells, CD11b^+^ macrophages infiltrating the dermis, and of the keratinocyte proliferation marker Ki67 (Figure 5). Conversely, compartimentalization and expression of the marker of differentiation, such as KRT10, rather weak in the suprabasal layer epidermis of IMQ-treated mice, were restored by treatment with the drug (Figure 5). Of note, tofacitinib dramatically reduced the presence of phospho-STAT3 and phospho-STAT1 in the nucleus of epidermal cells of mouse skin in a dose-dependent manner (Figure 6(a)), with the higher drug concentration responsible of almost total disappearance of phospho-STAT3^+^ (~80% of reduction) and STAT1^+^ cells (~95% of reduction) in IMQ-treated mice.

Since IL-17- and IL-22-producing leukocytes are implicated in the pathogenic processes associated to IMQ-induced psoriasiform reactions (28–30), and keratinocytes are actively involved in recruiting these cells in lesional skin, we investigated tofacitinib effect on the presence of IL-17- and IL-22-producing cells into mouse skin and the expression of chemokines potentially involved in their recruitment. Immunohistochemistry and real-time PCR of IL-17A and IL-22, together with IFN-*γ* and TNF-*α* mRNA analyses (Figure 6(b)), showed that tofacitinib decreased the number of IL-17^+^ or IL-22^+^ cells in the IMQ-treated skin, consistently with a significant reduction of IL-17 and IL-22 mRNA levels. In contrast, neither IFN-*γ* nor TNF-*α* mRNA expression could be significantly influenced by tofacitinib application (Figure 6(b)). Of note, both IL-17^+^ and IL-22^+^ cell populations had mostly a macrophage/dendritic cell-like morphology, similar to that of CD11c- and CD11b-stained cells (Figure 5). Finally, we analysed the expression of keratinocyte-derived chemokines in mouse skin biopsies by real-time PCR analysis. A significant reduction of chemokines, such as CCL2, CXCL16, CCL20, and IL-6, was detected after the application of tofacitinib (Figure 6(c)). The effect of tofacitinib was not exerted on CXCL10 or other keratinocyte chemokines whose expression was strictly dependent on IFN-*γ* (Figure 6(c) and data not shown).

The effect of tofacitinib was dose-dependent (Figures 4–6), and no change in all the analysed markers was observed by treating mouse skin with tofacitinib alone (not shown).

### 3.6. Tofacitinib and SOCS1 or SOCS3 Impair the Same Molecular Pathways in IFN-*γ*- or IL-22-Activated Keratinocytes

**Figure 7:**
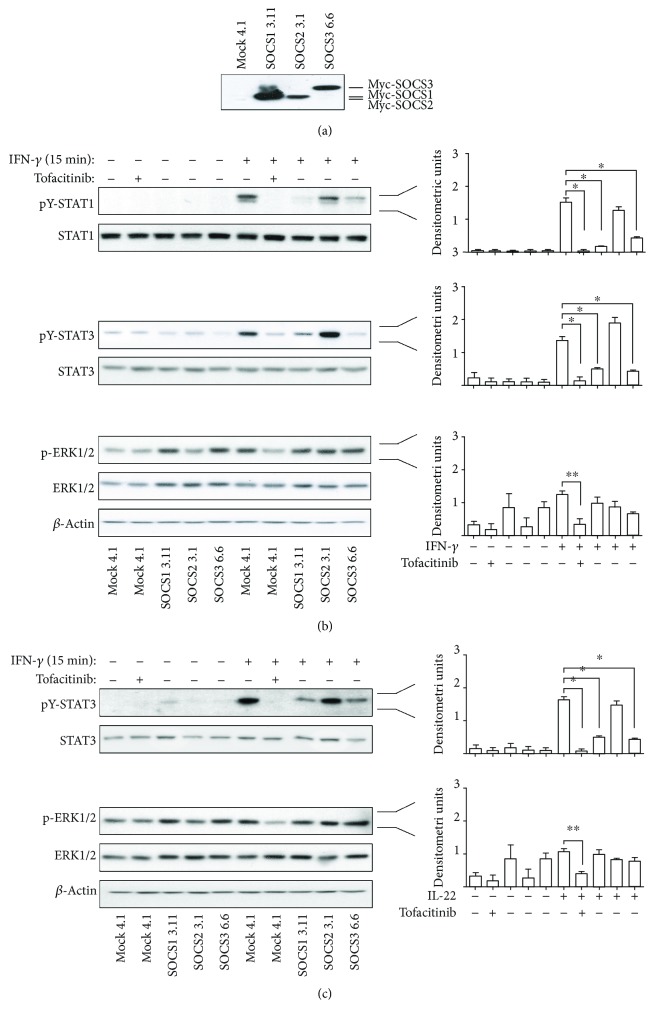
Tofacitinib inhibits the same IFN-*γ*- or IL-22-activated molecular pathways suppressed by SOCS1 or SOCS3. WB analysis was performed on protein lysates of HaCaT keratinocyte clones overexpressing SOCS1, SOCS2, or SOCS3, stimulated with IFN-*γ* (a) or IL-22 (b) or left untreated. Analysis was also performed on MOCK-transfected cells left untreated or treated with IFN-*γ* (a) or IL-22 (b), in the presence or absence of 5 *μ*M tofacitinib. Both basal and phospho-STAT1, phospho-STAT3, and phospho-ERK1/2 were evaluated. Graphs show densitometric analysis of WB bands, and data are expressed means of densitometric units, calculated using two and four different keratinocyte clones for each transgene and mock clones to detect STATs and ERK1/2 proteins, respectively. ^∗^*p* < 0.01, ^∗∗^*p* < 0.05.

In psoriatic keratinocytes, SOCS1 and SOCS3 molecules act as endogenous repressors of cytokine signaling and function by directly inhibiting JAK1 and JAK2 proteins, thus impeding STAT activation [13, 16]. This part of the study investigated whether tofacitinib could inhibit the same molecular pathways suppressed by SOCS1 and SOCS3 in keratinocytes. To this end, a comparison of the main molecular pathways activated by IFN-*γ* and IL-22 was carried out in keratinocyte overexpressing SOCS1 or SOCS3 and tofacitinib-treated mock clones. SOCS2 clones were used as negative control, as in these cells, IFN-*γ* or IL-22 signaling is not influenced by SOCS2 transgene presence [13]. A number of keratinocyte clones stably expressing SOCS were previously generated in our lab, as previously described [6, 13–15]. SOCS1, SOCS3, and SOCS2 clones (*n* = 2 for each) were tested for their levels of transgene contents and, then, activated with IFN-*γ* or IL-22 (Figure 7(a)). In parallel, mock clones (*n* = 2) were treated with tofacitinib together with IFN-*γ* or IL-22. The expression pattern of phosphorylated STAT1, STAT3, and ERK1/2 in IFN-*γ*- or IL-22-treated mock clones was identical to that induced in psoriatic keratinocytes, with tofacitinib efficiently inhibiting these signal transduction pathways (Figures 7(b) and 7(c)). Similarly to tofacitinib, the presence of SOCS1 or SOCS3 transgene determined an impairment of STAT1 and STAT3 activation in response to IFN-*γ*, and of STAT3 in response to IL-22, respectively (Figures 7(b) and 7(c)). Finally, the finding that phospho-ERK1/2 was efficiently inhibited in mock clones by tofacitinib was consistent with the absence of its upregulation in IFN-*γ*- or IL-22-treated SOCS1 and SOCS3 clones (Figures 7(b) and 7(c)). As expected, SOCS2 could not regulate molecular pathways triggered by IFN-*γ* and IL-22 in keratinocyte clones.

As a whole, these data demonstrate that tofacitinib similarly to SOCS1 and SOCS3, by targeting JAKs, can impair the same intracellular cytokine-dependent pathways in keratinocytes.

## 4. Discussion

Increasing evidence suggests that JAK proteins are a potential target for immunosuppressive drugs against psoriasis and other immune-mediated skin diseases, especially those elicited by epidermal keratinocytes exposed to massive amounts of proinflammatory cytokines, including IFN-*γ* and IL-22 [15, 16, 30]. In recent years, small molecule JAK inhibitors have been developed and extensively investigated for different pathological conditions [22, 24, 30]. Among them, the JAK inhibitor tofacitinib was shown to improve clinical outcomes in patients with moderate-to-severe psoriasis [19, 21, 24]. A recent study on its effects in psoriatic patients showed a dramatic and rapid shutdown of phospho-STAT1 and phospho-STAT3 and downstream-regulated genes in the epidermis and reduced pathologic T-cell and dendritic cell number in lesional skin, as well as expression of IL-17, IL-22, and IFN-*γ* [24].

The present study was aimed at understanding which inflammatory molecular pathway(s) activated can be specifically inhibited by tofacitinib in psoriatic keratinocytes. We found that this drug totally abrogated JAK/STAT pathways activated by IFN-*γ* and IL-22, as evaluated in experimental *in vitro* and *in vivo* models of psoriasis. These findings are important since IFN-*γ* and IL-22 inflammatory cytokines are deeply involved in the pathogenesis of psoriasis, as they stimulate keratinocyte proliferation, impair their differentiation, and promote a “feed-forward” inflammatory responses. Tofacitinib inhibition was exerted specifically on JAK1 and JAK2, but not on TYK2, and, as consequence, IFN-*γ* and IL-22 receptor phosphorylation, as well as the proximal cytokine signaling leading to STAT1 and STAT3 phosphorylation, were impaired. These effects were specific for IFN-*γ* and IL-22 and could not be observed on TNF-*α* or IL-17 signaling. This was not surprising, since TNF-*α* and IL-17 do not signal intracellularly through JAK/STAT and activate molecular pathways involving TRAF2/TRADD/NF-*κ*B or TRAF2/TRADD/MAPK and Act1/TRAF6/NF-*κ*B [11, 12]. In contrast, we observed that TNF-*α* induced STAT3 activation in keratinocytes, an effect that was partially inhibited by tofacitinib. This result could be explained by a direct action of tofacitinib on JAK-dependent signaling activated by TNF-*α*-induced cytokines (for instance IL-6), which could in turn activate STAT3 in an autocrine loop. Interestingly, tofacitinib also inhibited phosphorylation of ERK1/2 induced by IFN-*γ* or IL-22, but not that promoted by TNF-*α*. This dichotomy could depend by the fact that ERK1/2 activation by IFN-*γ* or IL-22 is mediated by JAK, whereas TNF-*α*-driven phosphorylation of ERK1/2 is downstream to TRAF2/TRADD [12].

A number of dysfunctional intracellular signaling pathways have been found in psoriatic keratinocytes other than STAT1 and STAT3, including NF-*κ*B-, AP-1-, p38-, and ERK1/2 kinase-activated pathways [4]. An analysis of additional intracellular kinases and signaling node molecules demonstrated that tofacitinib also reduces the IFN-*γ*-dependent upregulation of Akt, AMPK*α*, p38, PARP, and caspase 3 and the IL-22-dependent Akt, AMPK*α*, mTOR, HSP27, p38, and JNK. However, tofacitinib inhibitory effects on these molecular pathways were minimal if compared to those observed on STAT1 and STAT3, indicating an ancillary or indirect action of JAK in upregulating such pathways. Again, TNF-*α*-induced intracellular kinase pattern could not be influenced by tofacitinib, apart from those pathways that were dependent on JAK and not by TRAF2/TRADD.

Another part of the study intended to evaluate the effects of tofacitinib on those biological processes that are profoundly altered in psoriatic epidermis (i.e., proliferation, differentiation, and apoptosis) as a consequence of the deleterious effects of IFN-*γ* and IL-22 [6, 17]. In this context, we demonstrated that tofacitinib reduced proliferation and dedifferentiation promoted by IL-22 in keratinocytes. These results were confirmed in the IMQ *in vivo* murine psoriasis model, in which epidermal hyperproliferation, altered differentiation, and inflammation were mainly IL-22/STAT3-dependent [29]. The concurrent treatment with tofacitinib led to reduced expression of epidermal STAT3, proliferation markers, and increased production of markers of differentiation. Interestingly, tofacitinib also counteracted the cytostatic and proapoptotic effects of IFN-*γ* on keratinocytes, likely *via* inhibition of STAT1, known to mediate these effects. However, inhibition of IFN-*γ*-dependent antiproliferative effects on keratinocytes might not be strategic in a hyperproliferative disorders, such as psoriasis, although IFN-*γ* can induce massive proliferation of psoriatic stem cells, and its injection into prelesional psoriatic skin causes epidermal hyperplasia and plaque development [31, 32]. IFN-*γ* signaling and type 1T cells were shown to participate to the expression of psoriasiform phenotype in IMQ mice only partially [28]. Nonetheless, we found phospho-STAT1 localized in the nuclei of epidermal keratinocytes of IMQ-treated skin, with tofacitinib totally inhibiting its expression. It is plausible that inhibition of STAT1, together with STAT3, is indirectly responsible for the reduction in inflammatory infiltrate, due to the decrease of STAT1- and/or STAT3-dependent gene expression of chemokines in keratinocytes, such as CXCL10, CXCL1, CXCL8, CCL2, and CCL5, and of immunomodulatory molecules, including ICAM-1 and MHC class I and II. As result, T-cell, neutrophil, dendritic cell, and macrophage subpopulations could no longer accumulate in IMQ-treated skin in the presence of tofacitinib. Also IFN-*γ*-induced, but not IL-22-induced IL-6 and IL-20, two psoriasis-related cytokines were inhibited by this drug. It is noteworthy that the majority of inflammatory molecules induced by IL-22 in keratinocytes could not be downregulated by tofacitinib, with the exception of CX3CL1 and CXCL8 chemokines. Also, IL-22-dependent antimicrobial molecules could not be influenced. In contrast, SOCS3 mRNA expression was totally abrogated by tofacitinib, accordingly with our previous findings that STAT3-silenced keratinocytes were not able to upregulate SOCS3 in response to IL-22 [17].

Importantly, IL-17- and IL-22-producing cells were strongly reduced by JAK blockade in IMQ-treated skin, similarly to what observed in human psoriasis, where improvement of clinical and histologic signs by tofacitinib was associated with an inhibition of IL-17 gene expression and IL-23/Th17 pathway [24]. In contrast, neither IFN-*γ* nor TNF-*α* mRNA expression was influenced by tofacitinib. Immunohistochemistry analysis also showed that both IL-17^+^ and IL-22^+^ cells present in mouse skin had mostly a macrophage/dendritic cell-like morphology, accordingly with recent findings showing the presence and pathogenicity of IL-23-bearing and IL-17/IL-22-producing macrophage and dendritic cell subpopulations in the IMQ model [33]. In parallel, tofacitinib determined a strong reduction of keratinocyte-derived chemokines involved in the recruitment of pathogenic leukocyte populations *via* CCR2 or CCR6, such as CCL2 and CCL20. Although tofacitinib potently downregulated chemokine expression in keratinocytes and, in turn, leukocyte recruitment into mice skin, it is likely that its effect could be explicated directly on type 17 and 22T-cell differentiation, by interfering with IL-23R signaling and subsequent IL-17/IL-22 induction [23]. However, it is important to highlight the limited presence of IL-17- and IL-22-producing cells with a T-cell-like morphology in mouse skin at 5 days of IMQ application.

Finally, we demonstrated that tofacitinib and SOCS, in particular SOCS1 and SOCS3, by targeting identical signaling molecules, or JAK1 and JAK2, can impair the same intracellular pathways in keratinocytes. In fact, STAT1, STAT3, and ERK1/2 were not upregulated in keratinocyte clones overexpressing SOCS1 or SOCS3 in response to IFN-*γ*, nor STAT3 and ERK1/2 in response to IL-22, similarly to tofacitinib that abrogated cytokine-induced STAT1, STAT3, and ERK1/2 in control clones. These results are due to the fact that both tofacitinib and SOCS1/3 act on JAK with a high degree of kinome selectivity and display the same final biochemical effects of JAK inactivation. In fact, tofacitinib as well as SOCS1/3 impede auto- and transphosphorylation of JAK, with the first blocking ATP binding site of JAK1-2-3 and competing with ATP [34], and SOCS1/3 by interacting with the –GQM-amino acidic residues of JAK, determinant for its binding to substrates [35]. Importantly, while tofacitinib interacts with all JAKs but not with TYK2, SOCS1 and SOCS3 can bind and inactivate JAK1, JAK2, and TYK2 but not JAK3. Evidence that tofacitinib and SOCS1/3 can have the same anti-inflammatory effects on keratinocytes also comes from our recent studies performed with two small peptides mimicking SOCS1 and SOCS3 and sharing kinase inhibitory regions critical for JAK1 and JAK2 inactivation. Similar to tofacitinib, these two peptido-mimetics were able to switch off the IFN-*γ*- and IL-22-dependent inflammatory/immune responses of keratinocytes in cutaneous disease contexts characterized by the presence of IFN-*γ*-releasing Th1 and IL-22-releasing Th22 infiltrate, such as psoriasis and squamous skin cell carcinoma, respectively [15, 16].

## 5. Conclusions

As a whole, our study demonstrated the selectivity and specificity of tofacitinib inhibitory action on intracellular molecular pathways dependent on IFN-*γ* and IL-22 in keratinocytes. The blockade of IFN-*γ*/JAK1/JAK2/STAT1/STAT3 and IL-22/JAK1/STAT3 pathways had the important consequence to inhibit the expression of many IFN-*γ*-dependent inflammatory genes, as well as restore proliferative and differentiation programs altered by IL-22 in psoriatic keratinocytes. Considering that epidermal keratinocytes are the outermost component of the skin and that tofacitinib has a potent inhibitory effect on inflammatory responses evoked by these cells, it could be included in formulations for the topical therapy of psoriasis. Application of JAK inhibitors could be useful especially during the chronicization of the disease, where IFN-*γ*-dependent T-cell responses predominate. Indeed, the efficacy of topical therapy of tofacitinib and other JAK inhibitors in psoriasis has been extensively demonstrated [36] and is also considered for the treatment of other inflammatory skin conditions characterized by JAK hyperactivation, such as lichen planus and atopic dermatitis [37].

Importantly, tofacitinib inhibitory activity could also be explicated directly on type 17 and type 1T-cells, by impeding their differentiation and expansion. In fact, Th17 cell differentiation is abrogated in the absence of STAT3, whereas overexpression of a constitutively active STAT3 form results in greatly increased numbers of IL-17-producing cells [38, 39]. Similarly, STAT1 is abundantly activated in Th1 cells, mainly in response to IFN-*γ*, which is in turn critical to the generation and maintenance of Th1 immunity [23].

Due to the heterogeneity of pathogenic mechanisms operating in psoriasis, and to the variety of molecular cascades potentially activated by proinflammatory cytokines, a combination of JAK inhibitors and TNF-*α* or IL-17 blockers might elicit more favorable and efficacious therapeutic effects in psoriatic patients, by intercepting and blocking inflammatory responses at multiple levels.

Finally, local or systemic JAK/STAT inhibition by tofacitinib could be crucial for the development of optimized therapeutics also for the treatment of skin tumors characterized by aberrant IL-22 signaling and STAT3 activation in keratinocytes. The latter includes basalioma and squamous cell carcinoma, where IL-22-producing T cells aberrantly activate tumor growth and epithelial carcinogenesis through STAT3 (16).

## Figures and Tables

**Table 1:** Tofacitinib effects on the expression of inflammatory molecules induced by IFN-*γ* in keratinocytes.

	Untreated^∗^	TOF	IFN-*γ*	IFN-*γ* + TOF
Membrane molecules				
ICAM1	2.4 ± 0.2	2.0 ± 0.18	75.5 ± 6.3	6.8 ± 0.56†
HLA-DR	1.4 ± 0.15	1.4 ± 0.12	3.5 ± 0.25	1.4 ± 0.13†
MHC-I	72 ± 5.2	63 ± 4.5	146 ± 11.2	84 ± 7.4†
Chemokines				
CX3CL1	0.40 ± 0.05	0.36 ± 0.08	580.64 ± 55.06	26.20 ± 3.26†
CXCL1	0.80 ± 0.12	0.80 ± 0.07	69.00 ± 5.6	0.40 ± 0.08†
CXCL8	0.92 ± 0.11	2.60 ± 0.32	84.52 ± 7.45	5.04 ± 0.61†
CXCL10	29.08 ± 2.2	25.04 ± 0.14	2557.44 ± 150.2	29.08 ± 2.58†
CXCL12	107.20 ± 9.72	110.20 ± 2.43	141.52 ± 12.25	119.12 ± 10.61
CXCL16	2.92 ± 0.32	5.28 ± 0.68	18.48 ± 1.58	6.16 ± 0.81
CCL1	0.80 ± 0.1	0.80 ± 0.14	37.04 ± 2.94	0.60 ± 0.04†
CCL2	0.80 ± 0.95	0.76 ± 0.67	474.60 ± 49.46	0.80 ± 0.12†
CCL5	2.0 ± 0.4	1.9 ± 0.24	2305 ± 250.12	4.5 ± 0.6†
MIF	337.08 ± 35.71	637.04 ± 52.47	1745.88 ± 153.5	918.04 ± 85.8†
Cytokines, AMPs, SOCS				
IL-6	5.92 ± .0.79	5.28 ± 0.48	17.40 ± 1.57	5.28 ± 0.65†
IL-20	1.00 ± 0.12	0.90 ± 0.10	0.98 ± 0.10	0.58 ± 0.04†
LL-37	1.00 ± 0.11	0.89 ± 0.09	0.85 ± 0.10	0.70 ± 0.08
HBD2	1.00 ± 0.12	0.66 ± 0.08	1.11 ± 0.12	0.67 ± 0.08
S100A7	1.00 ± 0.13	1.11 ± 0.2	2.61 ± 0.0.25	2.83 ± 0.3
SOCS3	1.00 ± 0.12	1.13 ± 0.10	25.59 ± 2.65	2.14 ± 0.20†

Note: IFN: interferon; TOF: tofacitinib; ICAM: intercellular adhesion molecule; HLA-DR: human leukocyte antigen-antigen D related; MHC: major histocompatibility complex; CXCL: CXC-chemokine ligand; CL: chemokine ligand; MIF: macrophage migration inhibitory factor; IL: interleukin; LL37: antimicrobial peptide; HBD: human-defensin; S100: S100 calcium-binding protein; SOCS: suppressor of cytokine signaling. ^∗^Keratinocyte cultures were left untreated or treated with 5 *μ*M of tofacitinib and stimulated or not with 100 U/ml of IFN-*γ*. After 6 hours, IL-20, LL-37, HBD2, S100A7, and SOCS3 mRNA levels were analysed by real-time PCR and normalized to *β*-actin mRNA levels. Results are expressed as mean 2^−ΔΔCT^ ± SD. After 24 hours, cells were stained with ICAM1, HLA-DR, and MHC-I mAb followed by FITC-conjugated anti-mouse IgG and then analysed by flow cytometry. Data are expressed as mean ΔMFI ± SD. At the same time, supernatants were collected and, chemokines and IL-6 were measured by Bioplex, except for CCL5 which has been evaluated by ELISA. Results are expressed as mean pg/ml ± SD. †*p* < 0.05 compared to untreated or stimulated keratinocytes.

**Table 2:** Tofacitinib effects on the expression of inflammatory molecules induced by IL-22 in keratinocytes.

	Untreated^∗^	TOF	IL-22	IL-22 + TOF
Membrane molecules				
ICAM1	2.4 ± 0.2	2.0 ± 0.18	2.5 ± 0.24	1.8 ± 0.16
HLA-DR	1.4 ± 0.15	1.4 ± 0.12	1.4 ± 0.12	1.4 ± 0.11
MHC-I	72 ± 5.2	63 ± 4.5	57 ± 4.8	54 ± 5.2
Chemokines				
CX3CL1	0.40 ± 0.05	0.36 ± 0.08	46.72 ± 4.57	19.72 ± 1.87†
CXCL1	0.80 ± 0.12	0.80 ± 0.07	0.80 ± 0.09	0.40 ± 0.035
CXCL8	0.92 ± 0.11	2.60 ± 0.32	83.04 ± 9.43	49.04 ± 4.85†
CXCL10	29.08 ± 2.2	25.04 ± 0.14	36.60 ± 3.57	43.28 ± 3.19
CXCL12	107.20 ± 9.72	110.20 ± 2.43	119.12 ± 12.91	111.16 ± 5.76
CXCL16	2.92 ± 0.32	5.28 ± 0.68	12.92 ± 1.39	10.52 ± 1.15
CCL1	0.80 ± 0.1	0.80 ± 0.14	10.60 ± 1.08	10.60 ± 1.10
CCL2	0.80 ± 0.95	0.76 ± 0.67	1.72 ± 0.08	0.72 ± 0.62
CCL5	2.0 ± 0.4	1.9 ± 0.24	1.95 ± 0.16	1.73 ± 0.13
MIF	337.08 ± 35.71	637.04 ± 52.47	1800.12 ± 105.9	1807.00 ± 135.7
Cytokines, AMPs, SOCS				
IL-6	5.92 ± .0.79	5.28 ± 0.48	7.24 ± 0.84	6.24 ± 0.54
IL-20	1.00 ± 0.12	0.90 ± 0.10	3.17 ± 0.17	3.16 ± 0.05
LL-37	1.00 ± 0.11	0.89 ± 0.09	0.84 ± 0.11	1.51 ± 0.13
HBD2	1.00 ± 0.12	0.66 ± 0.08	3.62 ± 0.39	3.96 ± 0.56
S100A7	1.00 ± 0.14	1.11 ± 0.2	1.73 ± 0.16	1.71 ± 0.18
SOCS3	1.00 ± 0.12	1.13 ± 0.10	8.12 ± 0.95	0.64 ± 0.05

Note: IL: interleukin; TOF: tofacitinib; ICAM: intercellular adhesion molecule; HLA-DR: human leukocyte antigen-antigen D related; MHC: major histocompatibility complex; CXCL: CXC-chemokine ligand; CL: chemokine ligand; MIF: macrophage migration inhibitory factor; LL37: antimicrobial peptide; HBD: human-defensin; S100: S100 calcium-binding protein; SOCS: suppressor of cytokine signaling. ^∗^Keratinocyte cultures were left untreated or treated with 5 *μ*M of tofacitinib and stimulated or not with 75 ng of IL-22. After 6 hours, IL-20, LL-37, HBD2, S100A7, and SOCS3 mRNA levels were analysed by real-time PCR and normalized to *β*-actin mRNA levels. Results are expressed as mean 2^−ΔΔCT^ ± SD. After 24 hours, cells were stained with ICAM1, HLA-DR, and MHC-I mAb followed by FITC-conjugated anti-mouse IgG and then analysed by flow cytometry. Data are expressed as mean ΔMFI ± SD. At the same time, supernatants were collected and, chemokines and IL-6 were measured by Bioplex, except for CCL5 which has been evaluated by ELISA. Results are expressed as mean pg/ml ± SD. †*p* < 0.05 compared to untreated or stimulated keratinocytes.

## Data Availability

All the data used to support the findings of this study are included within the article, with the exception of data concerning (1) tofacitinib effects on IL-17 signal transduction in cultured keratinocytes; (2) tofacitinib effect on phospho-STAT1 expression, as detected by using phospho-kinase array kit; (3) tofacitinib effects on TNF-alpha- or IL-17-induced expression of inflammatory molecules by keratinocytes; and (4) tofacitinib effect on CD11c^+^ dendritic cells infiltrating the dermis in IMQ-treated mouse skin. The latter data are available from the corresponding author upon request.
